# The Impact of Cardio-Renal-Metabolic Profile in Dilated Cardiomyopathy

**DOI:** 10.1007/s11886-025-02241-8

**Published:** 2025-05-23

**Authors:** Tamim Akbari, Daniel J. Hammersley, Clarice Yau-Yee May, Brian P. Halliday, Sanjay K. Prasad

**Affiliations:** 1 Royal Brompton and Harefield Hospitals, Part of Guy’s and St Thomas’ NHS Foundation Trust, London, UK; 2 National Heart and Lung Institute, Imperial College London, London, UK; 3 King’s College Hospital, NHS Foundation Trust, London, UK; 4 CMR Unit, Royal Brompton Hospital, Sydney Street, London, SW3 6NP UK

**Keywords:** Dilated cardiomyopathy, Heart failure, Metabolic syndrome, Obesity, Multimorbidity, Outcomes

## Abstract

**Purpose of Review:**

Dilated cardiomyopathy is an important contributor to heart failure burden worldwide. With an aging population and rising multimorbidity, in this review, we describe the prevalence of metabolic syndrome and renal failure in patients with dilated cardiomyopathy and focus on common underlying mechanisms, evaluate outcomes in these patients and highlight newer therapeutic strategies.

**Recent Findings:**

A significant proportion of patients with dilated cardiomyopathy has concomitant metabolic syndrome and renal disease. This combination of multimorbidity portends worse prognosis and often presents unique challenges in treatment given the complex interplay and shared pathophysiological pathways.

**Summary:**

Optimization of the cardio-renal-metabolic profile should be a key consideration in the management of patients with dilated cardiomyopathy. Therapeutic strategies targeting common pathophysiological pathways are needed in order to improve overall outcomes.

## Introduction

Heart failure (HF) is estimated to affect around 64 million patients worldwide with cardiomyopathies constituting 5.4 million cases [1]. Dilated cardiomyopathy (DCM), an important underlying cause of heart failure [2], is characterized by left ventricular dilatation and reduced systolic function in the absence of coronary disease or abnormal loading conditions. The true prevalence of DCM is likely underestimated and is thought to be around 1 in 250 [3, 4] making it one of the most common cardiomyopathies, carrying a 20% 5-year mortality [5, 6].

**Fig. 1:**
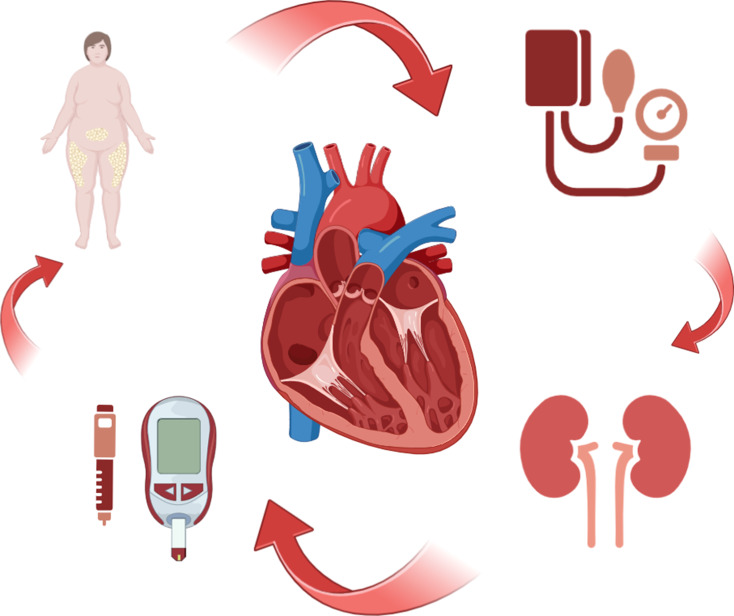
The impact of cardio-renal-metabolic profile in dilated cardiomyopathy. Metabolic syndrome and chronic kidney disease are common in dilated cardiomyopathy and carry worse prognosis. A complex interplay underpins common pathophysiological pathways. Therapies targeting these pathways are required to interrupt the cycle and improve outcomes in dilated cardiomyopathy. *Created in BioRender. Akbari*,* T. (2025)*https://BioRender.com/n26e844

Studies have shown the co-existence of metabolic syndrome, chronic kidney disease and heart failure suggesting shared pathophysiology and multi-directional interplay (Fig. 1) [7]. In patients with heart failure 20–30% have diabetes, 40–50% have chronic kidney disease and 8–16% have both, portending worse prognosis (Table 1) [8–10]. Given the interplay and to enable a holistic treatment approach, the term cardio-renal-metabolic syndrome has been proposed [7].

**Table 1:** Studies of outcomes in dilated cardiomyopathy with metabolic and renal disease

Study	Population (*n*)	Follow up (median /mean)	Comorbidities (% of patients)	Results/Conclusion
Eda et al., 2024	283 with DCM and 80 with NDLVC	68.8 months	DM = 21.5% HTN = 41.5% Dyslipidaemia = 32.9%	No difference in outcomes in DCM and NDLVC
Dziewiecka et al., 2023	617 DCM and 168 HNDC	47 months	DM = 22% HTN = 41.5% Dyslipidaemia = 65%	No difference in outcomes between DCM and HNDC
Silverdal et al., 2022	3733 DCM	50.4–60 months	DM = 11–20.8% HTN = 35.8–46.1% CKD = 1.7–3.7%	Diabetes associated with increased risk of outcomes (HR 1.35, 95% CI: 1.15–1.56; *p* = 0.0002)
EVITA-HF, 2020	323 (DCM with DM)	12.5 months	DM = 26.7% HTN = 78.9% CKD = 34.1%	Higher mortality in patients with DCM and diabetes as compared to DCM without diabetes (15.2% vs. 6.5%; *p* < 0.001)
Löfman et al., 2016	4939 DCM (total HF 47, 716)	Not available	DM = 32.3% HTN = 48.2% CKD = 51%	Increasing mortality with decreasing kidney function regardless of presence of diabetes and other factors
Karatolios et al., 2016	206 DCM	55.6 months	Not available Median values: BMI = 27.5 kg/m2 BP 120/77 mmHg eGFR = 79.9 ml/min/1.73 m	CKD associated with composite of all cause-mortality or heart transplantation (HR 2.42, 95% CI: 1.36–4.29; *p* = 0.003)
Österman et al., 2024	27,647 with CKD	Not available	CKD = 48% CKD + HF = 13% CKD + DM = 27% CKD + DM + HF = 13%	Highest all-cause mortality with CKD + DM + HF (HR 3.22, 95% CI: 3.05–3.39)

Abbreviations: *BMI* = body mass index; *BP* = blood pressure; *CKD* = chronic kidney disease; *DCM* = dilated cardiomyopathy; *DM* = diabetes mellitus; *eGFR* = estimated glomerular filtration rate; *HF* = heart failure; *HNDC* = hypokinetic non-dilated cardiomyopathy; *HTN* = hypertension; *NDLVC* = non-dilated left ventricular cardiomyopathy

### Metabolic Syndrome and Dilated Cardiomyopathy

Metabolic syndrome is a constellation of risk factors which includes hypertension, obesity, diabetes and dyslipidemia that collectively contribute to multiorgan damage, each represent risk factors for adverse cardiovascular outcomes. Inflammation and oxidative stress represent core pathophysiological drivers of disease in metabolic syndrome and are mediators of micro- and macrovascular damage, fibrosis, myocardial injury and adverse remodeling leading to accelerated coronary artery disease, cardiac arrhythmia and heart failure [11, 12].

In a recent retrospective study by Eda et al., 283 patients with DCM were evaluated with a median follow up of 68.8 months (Interquartile range; IQR 33-93.7 months). Metabolic risk factors were common as 21.5% had diabetes, 41.5% had hypertension and 32.9% had dyslipidemia [13]. Similar prevalence of diabetes (22%) and hypertension (47.3%) were reported in a larger study of patients with DCM by Dziewiecka et al. (*n* = 617) whereas dyslipidemia (65%) was present in a much larger proportion in this study [14]. In a large Swedish study of almost 1.7 million adolescent men (median follow up 27 years), obesity was strongly associated with the risk of DCM such that for each unit increase in body mass index (BMI), there was a 15% increase in risk (Hazard Ratio; HR per unit increase in BMI 1.15, 95% confidence interval (CI): 1.14–1.17) [15].

The presence of metabolic factors portends worse prognosis in heart failure [16, 17]. The same seems to hold true when the underlying etiology is DCM [18]. In a sub-analysis of the Candesartan in Heart failure-Assessment of Reduction in Mortality and morbidity (CHARM) program which randomized 7599 with symptomatic heart failure (diabetes = 28%, DCM = 13.6%), diabetes was associated with a greater relative risk of cardiovascular (CV) death or HF hospitalization irrespective of ejection fraction or underlying etiology [19]. In a large Swedish Registry study of recent and established DCM patients (*n* = 3733, median follow up 5 years), presence of diabetes was an independent predictor of major adverse cardiovascular events (MACE) and associated with a 34% increased relative risk of all-cause death, heart transplantation or HF hospitalization (HR 1.34, 95% CI: 1.15–1.56; *p* = 0.0002) as compared with DCM patients without diabetes [20]. Interestingly, the increase in mortality with diabetes in DCM is observed independently of ischaemic heart disease. In the large multicenter EVITA-HF registry (16 centres, *n* = 4,101), mortality in patients with diabetes and DCM (*n* = 323) was more than double (15.2%) that of DCM patients without diabetes (6.5%, *p* < 0.001, *n* = 885) [21]. This along with other studies suggest mechanisms other than accelerated coronary artery disease as the driver of outcomes [16]. Similarly, there is also evidence emerging for a causative role for type 1 diabetes leading to DCM, irrespective of obesity or hypertension status [22].

### The Obesity Paradox

The observation that patients with obesity (as measured by BMI) and HF (in particular with reduced LV systolic function) show better short to mid-term outcomes as compared to leaner patients is termed the obesity paradox [23]. This effect is counter intuitive as obesity leads to adverse cardiac remodeling and reduced metabolic efficiency, in particular in dilated cardiomyopathy [24, 25].

A number of studies have questioned the presence of a paradox [26–28] in particular, given in earlier studies, BMI has been used as a measure of adiposity which has several limitations. BMI does not accurately capture the extent of visceral fat relative to muscle mass nor does it differentiate retained fluid in decompensated HF from flesh weight. Moreover, these studies have not incorporated exercise capacity or other measures of cardio-respiratory fitness. In an analysis of the PARADIGM-HF study (*n* = 8,389), when waist to height ratio which is more likely to accurately capture central obesity, rather than BMI, was used to assess outcomes, no survival-paradox was observed [28]. Similarly, in a paediatric population of patients with DCM (*n* = 904, 13.3% obese), obesity did not confer a survival benefit [27]. Earlier studies reporting the paradox were marred by confounders and collider bias. Mendelian randomisation studies suggest a causal relationship between obesity and heart failure [29]. A growing body of evidence has convincingly demonstrated no survival benefit from obesity in HF when alternative anthropometric measures which more accurately capture the degree of adiposity are employed. Variation in adiposity measures are likely to account for the discrepancies in reported outcomes.

### Metabolic Syndrome, Metabolomics and DCM

The failing heart is considered ‘starved’ of energy. Typically, in heart failure there is increased glycolysis as a compensatory response to reduced mitochondrial oxidative capacity. In DCM associated with metabolic syndrome, there is a metabolic switch with an overreliance on free fatty acid (FFA) oxidation leading to reduced myocardial glucose uptake and oxidation [30]. Although this is less efficient, the failing heart relies on FFAs to generate most of its energy. In an analysis of biopsies in end stage DCM patients, a paucity of intracardiac free fatty acids was observed suggesting a problem with FFA import impairing the failing heart in meeting its fuel demands [31]. Other studies have not replicated these findings [32, 33]. Balance between glucose and fatty acid oxidation may depend on drivers of heart failure. Obesity/metabolic syndrome with increased FFA availability may predispose the DCM heart to increase FFA oxidation. A distinct phenotype is seen with DCM resulting in inefficient oxidative metabolism, insulin resistance and inflammation [34]. Further larger scale studies are required to untangle the complexities of energy metabolism in DCM.

The use of metabolomic technologies has opened avenues of research in the discovery of biomarkers for the diagnosis, response to treatment and prognosis of DCM [35, 36]. Even though common metabolomic perturbations are seen in heart failure of various etiologies, distinct profiles are observed in DCM as compared with heart failure as a result of other pathologies such as ischaemic (ICM) and valvular disease, with disease specific metabolomic signatures [37]. In one recent study of 65 patients with acute and chronic heart failure (DCM = 25); 630 plasma metabolites were analyzed. Metabolites of fatty acid metabolism were affected in both DCM and ICM compared with control, emphasizing general abnormalities of lipid metabolism and lipo-toxicity in patients with HF. However, of the total, 30 metabolites were altered in DCM as compared to controls and 8 metabolites (serine, lysophosphatidylcholine, cholesteryl esters [16:0, 18:1, 18:3, 20:3, 20:4, 22:5]) were altered when DCM was compared with ICM [38]. In another study, (DCM = 38, ICM = 18, healthy controls = 20) the metabolomic profile of plasma samples identified important dysregulated pathways. Alpha-linolenic acid metabolism was identified as a significant pathway in the DCM group whereas linoleic acid, D-Glutamine and D-glutamate metabolism were identified as significant pathways in the ICM group. The group also identified six metabolites (phosphatidylcholine 1-pyrroline-2-carboxylate, norvaline, lysophosphatidylinositol [16:0/0:0], phosphatidylglycerol [6:0/8:0], fatty acid esters of hydroxy fatty acid [24:1] and phosphatidylcholine [18:0/18:3] to be significantly different between patients with DCM and ICM [37]. These data suggest disease specific metabolomic perturbation, challenging the notion of a universal HF metabolomic signature. Limitations include small numbers of patients, lack of generalizability to wider ancestries and the use of animal models and explanted hearts for tissue metabolomic analysis. Adequately powered studies with both plasma and tissue analysis need to be conducted to explore the clinical potential of disease specific metabolomic signatures in the diagnosis and management of DCM.

### Cardio-Renal Syndrome in DCM

Cardio-renal syndrome encompasses a bidirectional pathology involving the heart and kidneys classified into several types based on which organ is felt to be the primary driver of dysfunction [39]. Both organs are intricately linked as the kidneys are reliant on the heart for adequate perfusion and in turn dictate volume status and therefore myocardial volume load. Poor renal perfusion due to heart failure leads to activation of the renin-angiotensin-aldosterone and sympathetic systems which in turn can lead to fluid retention and vascular congestion exacerbating cardiac output leading to a vicious spiral [40].

Many risk factors are shared by renal disease and heart failure and up to half of patients with heart failure have some degree of kidney failure [41, 42]. The reported incidence of chronic kidney disease in DCM varies ranging from 15 to 40% [43, 44]. Renal impairment is associated with worse clinical outcomes in DCM. Impaired renal function also may restrict pharmacological treatment options. In a large Swedish registry of heart failure patients (*n* = 47, 716), 17% of patients of at least moderate renal impairment (eGFR < 60 mL/min/1.73 m^2^) had DCM as the underlying heart failure etiology. Renal dysfunction was associated with mortality irrespective of age or presence of diabetes [45]. In a study of 206 patients with DCM (mean follow-up 55.6 ± 18.4months); renal impairment (GFR < 60 ml/min/1.73m^2^) was again identified as an independent predictor of the composite end point of all-cause mortality or heart transplantation (HR 2.42, 95% CI: 1.36–4.29; *p* = 0.003) [46].

### Impact of Concomitant Diabetes and CKD in DCM

In a recent study of primary care records linked with hospital episode statistics across general practices in England, heart failure (all causes) was present in 13.7% of the population with CKD and T2DM (*n* = 183,997). All-cause mortality (rate/1000-person years; 192.9, 95% CI: 188.8–197.0) and cardiovascular outcomes (mortality/hospitalization; 149.0, 95% CI: 145.4–152.7) were highest when all three were present. The study also reported low use of newer therapies in the cardio-renal-metabolic domain (Sodium-glucose co-transporter-2 (SGLT2) inhibitor use = 2.8%; Glucagon-like peptide-1 (GLP-1) use = 3.4%) although with a trend towards increasing use [47]. Similar results were reported in another study analyzing new onset heart failure (*n* = 87,709) using the same database. Patients with heart failure, CKD and T2DM constituted 16% of the population and the presence of CKD and T2DM in heart failure was associated with the worst median survival (ranging from 2. 8–0.7 years based on CKD stage) [8]. Whilst these and other studies report worse prognosis [48, 49], there is paucity of data on outcomes specific to dilated cardiomyopathy with co-existent diabetes and chronic kidney failure. In a recent Swedish national registry study (*n* = 27,647, prevalence of HF-T2DM-CKD = 13%), the etiology of heart failure was non-ischaemic in 49% of patients with HF, CKD and T2DM. All-cause mortality and MACE were highest when all three were present (HR for all-cause mortality; 3.22, 95% CI: 3.05–3.39) [50]. Larger studies are required to ascertain outcomes in DCM patients with multimorbidity to identify gaps in treatments in order to direct healthcare resources appropriately.

### Interaction Between Genetics, Sex and Cardio-Renal Metabolic Profile in DCM

The genetic architecture in DCM is of prognostic significance. A familial cause is implicated in 25–35% of cases and many pathological and likely pathological variants are associated with worse cardiovascular outcomes [51–53]. A study in 487 patients with DCM showed that carriers of desmosomal and lamin A/C (LMNA) genetic variants experienced a significantly higher rate of ventricular arrhythmias and sudden cardiac death (SCD), independent of ejection fraction [54]. Similarly, carriers of titin-truncating variants, the most common genetic cause of DCM, in certain settings, appears to increase the risk of life-threatening arrhythmias [55].

Interestingly, in a large study of 60,706 individuals, mutation in the LMNA gene (variant p.G602S) was shown to associate with type 2 diabetes [56]. Other mutations in LMNA genes have also been shown to associate with severe metabolic syndrome [57]. In one study, genes involved in lipid metabolism were able to predict the risk of DCM with good accuracy (AUC, training set 0.936; test set 0.737) [58]. In another study, authors analyzed the Gene Expression Omnibus (GEO) dataset to identify differentially expressed genes in obesity and DCM and showed that genes implicated in oxidative stress, metabolic disorders and fibrosis play roles in obesity induced DCM [59]. Further evidence linking obesity and DCM genetics was reported by Zheng et al. in a large genome wide association study. Authors developed a polygenic risk score for DCM risk in participants in the UK biobank (AUC 0.71) using common genetic variants (> 500,000 single nucleotide polymorphisms). The risk score was shown to associate with obesity amongst other cardiovascular diseases on phenome-wide association studies suggesting a causal relationship [60]. In totality, this body of evidence appears to suggest common genetic pathways between metabolic disease and DCM.

Biological sex has also been shown to impact the clinical course and outcomes in DCM. There is a preponderance of male sex in DCM studies with a 3:1 ratio to female patients. This imbalance is not explained by survival bias and suggests greater penetrance or detection in men. A number of studies have reported worse cardiovascular outcomes and all-cause mortality with male sex in DCM [61–65]. A meta-analysis of 5 studies (*n* = 5,709) showed higher all-cause mortality (HR 1.61, 95% CI: 1.36–1.90; *p* < 0.00001), SCD (HR 1.80, 95% CI: 1.63–2.61; *p* = 0.002) and cardiovascular mortality (HR 1.67, 95% CI: 1.25–2.23; *p* = 0.0005) in male patients with DCM as compared to female [64]. However, more recently this concept has been challenged. In a recent prospective study by our group (*n* = 604, 206 female, median follow up 3.9 years), despite a more favourable profile (less myocardial fibrosis, higher LV ejection fraction), the composite of cardiovascular mortality or major heart failure events was higher in female patients with DCM as compared to those of males with DCM (8.6% vs. 4.4%, adjusted HR 3.14, 95% CI: 1.55–6.35; *p* = 0.001) at two years suggesting worse prognosis in female patients early on in the disease trajectory despite a milder phenotype [66]. Further large scale studies are required to shed additional light on the role that biological sex plays in determining both short and longer-term outcomes in patients with DCM.

### Novel Therapeutic Directions

There is a need to develop therapies targeting the common pathophysiological pathways of DCM and metabolic-renal dysfunction. Trimetazidine (an inhibitor of long-chain 3-ketoacyl-CoA thiolase) inhibits oxidation of free fatty acids thereby shifting substrate utilization to glucose and improving cardiac metabolism. In a recent meta-analysis of 28 studies (*n* = 2552, median follow up 6 months) of patients with HF with reduced ejection fraction (non-ischaemic, *n* = 359), Trimetazidine was associated with significantly reduced cardiovascular mortality (Odds ratio; OR 0.33, 95% CI: 0.21 to 0.53) and HF hospitalizations (OR 0.42, 95% CI: 0.29 to 0.60) [67].

With the advent of SGLT2 inhibitors, intervention on a number of pathways, both related to diabetes and more likely common pro-inflammatory and pro-fibrotic pathways, has already impacted significantly on improving outcomes for patients with heart failure and DCM. Developed initially as glucose lowering agents in type 2 diabetes, a number of trials have shown beneficial effects of these agents in HF and CKD irrespective of diabetes status [68–71] The exact mechanisms by which they exert their effects in HF are unknown. Modulating key cellular mechanisms affecting inflammation is proposed as a key hypothesis. In one small observational study of patients with DCM (*n* = 50), Dapagliflozin use was associated with better cardiac function at 12 months as compared with conventional therapy without SGLT2 inhibitor use [70]. Similarly, the GLP-1 agonist, Semaglutide has recently been shown to improve outcomes in patients with HF of both preserved and reduced ejection fraction and obesity (irrespective of diabetes status) in the large randomized controlled SELECT trial [72]. Whether GLP1-agonists will also become a significant pillar of treatment in the management of DCM and HF with reduced ejection fraction in general remains to be determined [73, 74].

## Conclusions

Advances in the diagnosis and management of dilated cardiomyopathy have resulted in improvement in outcomes over the last two decades. However, an ageing population accumulating metabolic and renal comorbidities in DCM pose new challenges. The cardio-renal-metabolic profile is an important consideration in all patients with DCM and optimization should become a benchmark component of clinical care. Large scale studies are required to ascertain the role of biological sex, genetics and ancestry to fill gaps in knowledge in order that equitable health care is provided universally.

## Key References

- Ndumele CE, Neeland IJ, Tuttle KR, et al. A Synopsis of the Evidence for the Science and Clinical Management of Cardiovascular-Kidney-Metabolic (CKM) Syndrome: A Scientific Statement from the American Heart Association. Circulation. 2023;148:e183-e201.
-
  - This scientific statement from the American Heart Association summarizes the key considerations in the management of cardiovascular-kidney-metabolic syndrome in established cardiovascular disease.
- Eda Y, Nabeta T, Iikura S, et al. Non-dilated left ventricular cardiomyopathy vs. dilated cardiomyopathy: clinical background and outcomes. ESC Heart Fail. 2024;11:236–245.
-
  - This recent study following up patient with dilated cardiomyopathy reported metabolic risk factors to be commonly present in these patients.
- Österman J, Al-Sodany E, Haugen Löfman I, Barany P, Evans M. Heart failure: the grim reaper of the cardio‐renal‐metabolic triad. ESC Heart Fail. 2024;11:2334–2343.
-
  - In this study following up patients for up to 10 years, the concomitant presence of chronic kidney disease, diabetes and heart failure portended the worst prognostic outcomes.

## Data Availability

No datasets were generated or analysed during the current study.
